# New species of Cerambycidae (Coleoptera) from South and Central America

**DOI:** 10.3897/zookeys.530.6155

**Published:** 2015-10-28

**Authors:** Maria Helena M. Galileo, Antonio Santos-Silva, Stéphane Le Tirant

**Affiliations:** 1 PPG Biologia Animal, Departamento de Zoologia, Universidade Federal do Rio Grande do Sul, Porto Alegre, RS, Brazil (Fellow of the Conselho Nacional de Desenvolvimento Científico e Tecnológico); 2 Museu de Zoologia, Universidade de São Paulo, São Paulo, São Paulo; 3 Insectarium de Montréal, 4581 rue Sherbrooke est Montréal, Québec, Canada

**Keywords:** Longhorned beetle, Tropidina, taxonomy

## Abstract

Three new species are described: *Tropidion birai* (Cerambycinae, Neoibidionini) from Bolivia; *Chrysoprasis birai* (Cerambycinae, Heteropsini) from Panama; and *Recchia nearnsi* (Lamiinae, Aerenicini) from Bolivia. The new species are included in amended versions of previously published keys to species of each genus.

## Introduction

Currently *Tropidion* Thomson, 1867 encompasses 75 species, distributed mainly in South America, with only four species occurring in Central America (Monné 2015a). Martins and Galileo (2007) revised the South American species of *Tropidion* and provided a key to the species of that region. Accordingly, Neoibidionini is divided in three subtribes: Ibidionina Thomson, 1860 (currently, Neoibidionina Monné, 2012), Compsina Martins and Galileo 2007, and Tropidina Martins and Galileo 2007. Compsina has the procoxal cavities closed behind, while in Tropidina and Neoibidionina they are open behind. *Tropidion* is the type genus of Tropidina, a subtribe that differs from Neoibidionina by the scape piriform, usually with basal sulcus, and by the antennomeres III–V with subequal length. In Neoibidionina the scape is cylindrical, slightly and gradually thickened toward apex, and antennomere IV is shorter than III and V.

*Chrysoprasis* Audinet-Serville, 1834 includes 70 species from North (Mexico) to South America (Monné 2015a). Napp and Martins (1995, 1997, 1998, 1999) divided the genus in four groups of species: Group *basalis*, characterized by the elytra bicolorous or, when with a single color, the prothorax with integument bicolorous or totally orangish; Group *chalybea*, with elytra and/or prothorax with single metallic color, and the ventrites are black or have metallic color; Group *hypocrita*, with the ventrites reddish and pronotal and elytral integument with single metallic color; and Group *aurigena*, with the elytra with bands or maculae of metallic color cupreous, golden, blue or violaceous. The new species herein described belongs to the latter group.

The genus *Recchia* was described by Lane (1966) with a single species, *Recchia ludibriosa* Lane, 1966, from São Paulo (Brazil). Martins and Galileo (1985) revised *Recchia*, described eight new species, and transferred an additional eight species to the genus. Martins and Galileo (1998) revised Aerenicini, described three more new species in *Recchia*, and provided a key to the species of the genus. Monné (2015b) listed 22 species in *Recchia*, distributed most in South America (only *Recchia hirsuta* (Bates, 1881) occurring in Central America). Recently, Mehl et al. (2015) described a new species from Paraguay. Thus, with the new species herein described, currently *Recchia* has 24 species.

## Material and methods

Photographs were taken with a Canon EOS Rebel T3i DSLR camera, Canon MP-E 65mm f/2.8 1–5X macro lens, controlled by Zerene Stacker AutoMontage software. Measurements were taken in ‘‘mm’’ using a micrometer ocular Hensoldt/Wetzlar - Mess 10 in the Leica MZ6 stereomicroscope, also used in the study of the specimen.

The collection acronyms used in this study are as follows:

MNKM Museo de Historia Natural Noel Kempff Mercado, Santa Cruz, Bolivia;

MZSP Museum of Zoology of the University of São Paulo, São Paulo, Brazil;

USNM National Museum of Natural History, Washington, DC, USA.

## Systematics

### Neoibidionini Monné, 2012

#### Tropidion birai sp. n.

Taxon classificationAnimaliaColeopteraCerambycidae

http://zoobank.org/816A1A7B-73E1-45C6-8D54-CC8DCA9AC3EB

Figs 1 , 2 , 3 , 4

##### Description.

Male. Integument brown, except for: apex of mandibles black; parts of head reddish-brown; base of scape dark-brown, remaining surface orangish-brown; pedicel and antennomeres dark-brown, gradually lighter toward distal antennomeres (but with apex of antennomeres blackish); two large, yellowish maculae on each elytron; most of peduncle of femora dark-brown; most femoral club orangish-brown; apex of femoral club brownish; tibiae dark-brown, except for reddish area inside of longitudinal sulcus; ventrites orangish-brown.

Head. Frons punctate-rugose; with short, moderately abundant setae, not obliterating integument. Area between antennal tubercles and eyes microsculptured, interspersed with coarse, sparse punctures. Area between posterior ocular edge and prothorax moderately coarsely, abundantly punctate (punctures denser near prothorax). Area behind lower eye lobes with punctures slightly distinct; with long, sparse setae close to eye; area under lower eye lobes with short, moderately abundant, yellowish-white setae. Area between anterior emargination of eyes and antennal fovea with short, dense yellowish-white pubescence. Genae moderately coarsely, abundantly punctate; with short setae interspersed with moderately long, sparse setae. Coronal suture distinct from clypeus to level of posterior ocular edge. Antennal tubercles notably projected upward, in frontal view, horn-like. Distance between upper eye lobes 0.55 times length of scape; distance between lower eye lobes 1.2 times length of scape in ventral view, 0.8 times length of scape in frontal view. Submentum not well-delimited, transversely striate, moderately coarsely, abundantly punctate; with short, moderately abundantly setae (not obliterating integument), interspersed with long, sparse setae. Antennae as long as 3.0 times elytral length; reaching elytral apex at basal quarter of antennomere VII; scape dorsally with distinct sulcus from base to about middle, with short, sparse setae, interspersed with very long setae; antennomere III dorsally longitudinally sulcate, pubescent, with fringe of long setae on inner lateral side; remaining antennomeres pubescent, with fringe of setae on inner lateral side gradually sparser; antennal formula (ratio) based on antennomere III: scape = 0.52; pedicel= 0.18; IV = 0.94; V = 1.11; VI = 1.15; VII = 1.02; VIII = 1.02; IX = 1.02; X = 0.82; XI = 1.39.

Thorax. Prothorax cylindrical, 1.7 times as long as wide; basal and distal sixth somewhat enlarged. Pronotum finely, sparsely punctate, except for basal sixth, where punctures are slightly coarser, distinctly denser; with five tubercles: one, subrounded, on each side of basal third; one, subrounded, on each side just after middle; one, subconical, on center; one each side, with wide band with yellowish-white pubescence, wider at base, that does not reach anterior margin; remaining surface with short, very sparse setae, interspersed on distal half by long setae. Prothorax laterally glabrous, finely, sparsely punctate, except for anterior sixth with transverse, fine striae. Prosternum finely, transversely striate on anterior half; each side of basal half with band with yellowish-white pubescence, divergent, narrowed from procoxal cavity to just after middle. Prosternal process longitudinally sulcate (mainly on distal half), pubescent; distal portion subcordiform. Mesosternum without tubercle, almost glabrous centrally, pubescent laterally (pubescence not obliterating integument). Mesepisterna and mesepimera densely pubescent (obliterating integument). Metasternum laterally and posteriorly pubescent (not obliterating integument); remaining surface with moderately sparse pubescence, except for glabrous central area near mesocoxal cavities. Metepisterna densely pubescent (partially obliterating integument). Scutellum densely pubescent. Elytra sparsely punctate, nearly all with a moderately long, yellowish setae (on circum-scutellar region punctures are blackish, part of them asperate-like); apical margin concave, with long spine at outer angle and projected at sutural angle. Legs. Femora clavate, very finely pubescent, with long, sparse setae; apex rounded on both sides. Tibiae longitudinally sulcate on both sides. Metatarsomere I as long as II–III together.

Abdomen. Ventrites I–IV pubescent laterally, centrally with short, sparse setae, interspersed with some long setae. Ventrite V with short, very sparse setae on basal third; distal two-thirds pubescent, interspersed with long setae; apex truncate, slightly emarginate.

**Figures 1–8.**
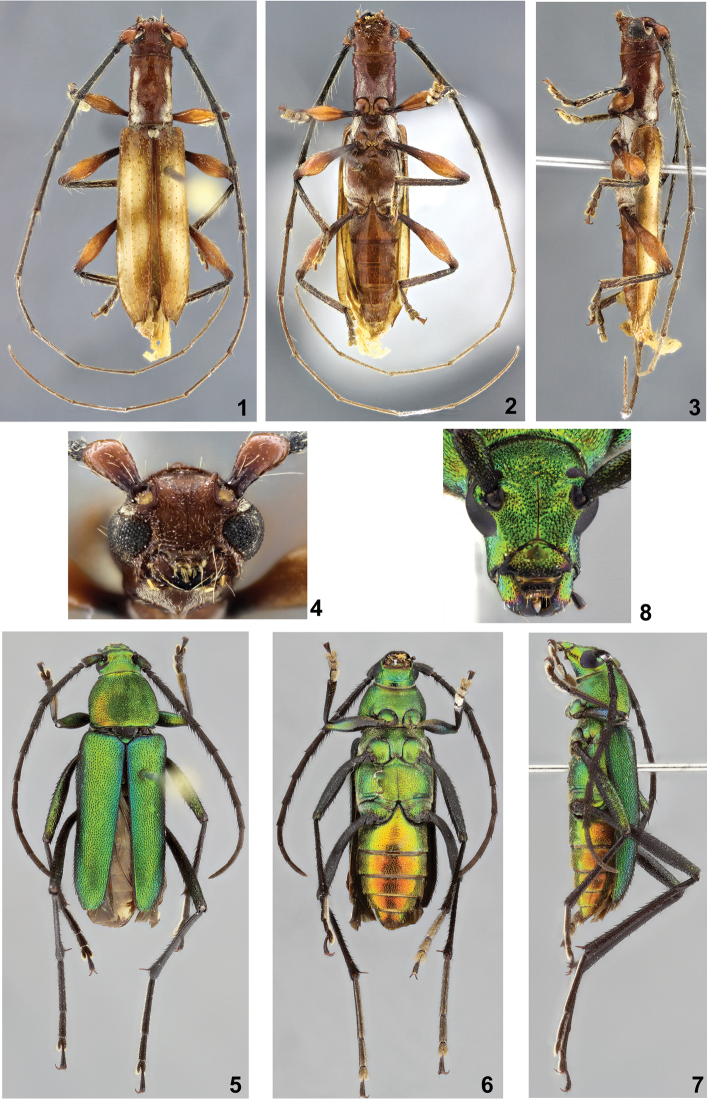
**1–4** *Tropidion birai* sp. n., holotype male: **1** Dorsal habitus **2** Ventral habitus **3** Lateral habitus **4** Head, frontal view **5–8** *Chrysoprasis birai* sp. n., holotype female: **5** Dorsal habitus **6** Ventral habitus **7** Lateral habitus **8** Head, frontal view.

##### Dimensions in mm

**(holotype male)**. Total length (from mandibular apex to abdominal apex), 11.80; prothorax: length, 2.70; anterior width, 1.65; posterior width, 1.70; humeral width, 2.30; elytral length, 7.70.

##### Type material.

Holotype male from BOLIVIA, Yungas, 1-28.VI.2005, Y. Callegari col. (MZSP).

##### Etymology.

The new species is named after the late Ubirajara Ribeiro Martins de Souza (Bira).

##### Remarks.

*Tropidion birai* sp. n. is similar to *Tropidion centrale* (Bates, 1872), a species very variable in color, but differs mainly by the pronotum with longitudinal bands of pubescence and with distinct tubercles. *Tropidion centrale* has pubescence on pronotum only on narrow transverse basal band, and does not have distinct tubercles on pronotum. From *Tropidion abditum* Martins, 1968 by the pronotum with bands of pubescence (not pubescent in *Tropidion abditum*), and by the apex of elytra with distinct spine at outer angle (unarmed in *Tropidion abditum*). It differs from *Tropidion bituberculatum* (Audinet-Serville, 1834) by the antennal tubercles acute at apex (not so in *Tropidion bituberculatum*), by the inner side of antennomere III with abundant and long setae (short and sparse in *Tropidion bituberculatum*), and by the central tubercle of pronotum very distinct (slightly distinct in *Tropidion bituberculatum*). It can be separated from *Tropidion buriti* Martins & Galileo, 2012 by the pronotum with distinct tubercles (slightly conspicuous in *Tropidion buriti*) and with bands of pubescence (without bands of pubescence in *Tropidion buriti*), and by the apex of elytra with distinct spine at outer angle (unarmed in *Tropidion buriti*). From *Tropidion carinicolle* (Bates, 1872), *Tropidion intermedium* (Martins, 1962), and *Tropidion flavum* (Martins, 1962) it differs mainly by the presence of yellowish maculae on elytra (absent in those species). *Tropidion birai* differs from *Tropidion festivum* (Martins, 1962) and *Tropidion validum* (Martins, 1962), by the basal antennomeres distinctly dark (uniformly reddish in those species), by the elytral maculae distinctly large, mainly the posterior. It differs from *Tropidion jolyi* Martins & Galileo, 2012 by the antennomere III distinctly carinate in male (not so in *Tropidion jolyi*), and by the presence of bands of pubescence on pronotum (absent in *Tropidion jolyi*). It differs from *Tropidion hermione* (Thomson, 1867) mainly by the pronotum with bands of pubescence (absent in *Tropidion hermione*). From *Tropidion mirabile* Martins, 1971 and *Tropidion praecipuum* Martins, 1971 it differs by the posterior yellowish band of elytra oblique to the suture (transverse in those species).

*Tropidion birai* can be included in the alternative of couplet “70”, from Martins and Galileo (2007) (translated):

**Table:** 

70(66)	Basal antennomeres dark-brown; posterior yellowish macula of the elytra wide. Bolivia	***Tropidion birai* sp. n.**
–	Antennomeres reddish; posterior yellowish macula of the elytra narrow	**70**’
70’(70)	Prothorax narrower at level of posterior third; pronotum shiny, with central tubercle longitudinal and well-marked; punctures near scutellum asperous. Bolivia	***Tropidion festivum* (Martins, 1962)**
–	Prothorax with basal constriction slightly notable; pronotum opaque, microsculptured (32 ×), and central tubercle less distinct; punctures near scutellum not asperous. Bolivia, Paraguay	***Tropidion validum* (Martins, 1962)**

#### Chrysoprasis birai sp. n.

Taxon classificationAnimaliaColeopteraCerambycidae

http://zoobank.org/AC84D640-87A6-46AB-A42A-E3C720E92910

Figs 5 , 6 , 7 , 8

##### Description.

Female. Integument metallic green with golden reflex; elytra darker, without golden reflex, with narrow violaceous band on base and along suture (slightly wider on basal two-thirds of suture), and wide violaceous band laterally (less conspicuous depending on angle of incidence of light; anteclypeus most reddish; apex of labrum reddish; ventrites cupreous, with violaceous and green reflexes, more distinctly violaceous on ventrite V; scape with distinct violaceous reflexes, more distinct depending on angle of incidence of light; antennomeres dark violaceous with some green reflexes (distal antennomeres more opaque); legs violaceous with green reflexes.

Head. Frons moderately finely, abundantly, confluently punctate, with short, decumbent, abundant setae (very slightly conspicuous). Coronal suture well-marked from clypeus to anterior level of eyes. Postclypeus with microsculptured, subtriangular area at base (interspersed with some coarse punctures); remaining surface moderately finely, abundantly punctate; punctate area with very short, slightly conspicuous setae, and one long seta on each side. Anteclypeus narrow. Labrum smooth at base, moderately finely, abundantly punctate on remaining surface; on punctate area with short, moderately abundant setae (mainly at distal third), interspersed with long setae. Antennal tubercles with sculpture as on frons. Area between antennal tubercles and upper eye lobes transversely sulcate. Vertex with sculpture as on frons. Area behind upper eye lobes with sculpture as on frons, gradually slightly sparser towards behind lower eye lobes (somewhat striate close to prothoracic margin). Genae moderately finely, abundantly punctate, punctures finer toward apex. Gulamentum transversely striate on narrow area closer to thorax (centrally smooth), coarsely vermiculate-punctate on large area closer to maxilla; this latter with short, moderately abundant setae, interspersed with long setae. Distance between upper eye lobes 0.85 times length of scape; distance between lower eye lobes, in frontal view, 1.05 length of scape. Antennae as long as 1.5 times elytral length; reaching elytral apex at middle of antennomere XI; scape coarsely, densely punctate, with long, thick, sparse, dark setae; antennomeres III–VI with short, but distinct spine at inner apex; antennomeres VI-X with distal outer distal angle projected; antennomere XI somewhat divided at distal third, almost forming twelfth segment; antennal formula (ratio) based on antennomere III: scape = 1.84; pedicel = 0.15; IV = 0.56; V = 0.64; VI = 0.66; VII = 0.66; VIII = 0.58; IX = 0.50; X = 0.43; XI = 0.61.

Thorax. Prothorax wider than long; lateral sides divergent from anterior margin to about apex of anterior third, subparallel toward posterior margin; anterior margin notably narrower than basal margin. Pronotum moderately coarsely, densely punctate, except for narrow, transverse, smooth band close to basal margin; with short, abundant, but slightly conspicuous setae, interspersed with some long setae laterally. Lateral sides of prothorax moderately coarsely, densely punctate (punctures slightly larger and shallower than on pronotum); setae as on pronotum. Prosternum finely, densely punctate throughout, with short, abundant setae. Prosternal process longitudinally sulcate, centrally narrowed; with sculpture and setae as on prosternum. Mesosternum and mesosternal process with sculpture and setae as on prosternum. Apex of mesosternal process strongly emarginate. Mesepisterna moderately finely, densely punctate. Mesepimera finely, abundantly punctate. Metepisterna moderately finely, densely punctate; with short, decumbent, abundant setae. Metasternum moderately finely, abundantly punctate throughout (punctures slightly finer toward center); with short, abundant, decumbent setae. Scutellum finely punctate laterally, longitudinally sulcate, smooth at center. Elytra. Surface microsculptured; moderately coarsely, densely punctate; with short, abundant, decumbent setae, interspersed with thick, dark setae, mainly at base and distal third; apex truncate, with outer angle projected and sutural angle rounded. Legs. Femora moderately finely densely punctate on peduncle, coarsely, densely punctate on club. Metatarsi slightly shorter than metatibiae; metatarsomeres I and II cylindrical; metatarsomere I notably elongate, 2.1 times longer than II.

Abdomen. Ventrites finely, abundantly punctate; with short, moderately abundant setae interspersed with long setae; apex of ventrite V rounded.

##### Type material.

Holotype female from PANAMA, *Panama*: Barro Colorado Island, 13–20.V.1998, Don Windsor col. (USNM).

##### Dimensions in mm

**(female).** Total length, 12.3; length of prothorax at center, 2.5; anterior width of prothorax, 1.9; posterior width of prothorax, 2.9; largest width of prothorax, 3.1; humeral width, 3.6; elytral length, 7.7.

##### Etymology.

The new species is named after the late Ubirajara Ribeiro Martins de Souza (Bira).

##### Remarks.

*Chrysoprasis birai* sp. n. differs from *Chrysoprasis quadrimaculata* Gounelle, 1913, and *Chrysoprasis suturalis* Lameere, 1884, mainly by the prothorax wider than long (about as long as wide in those species), and by the spine of the antennomeres III–VI distinctly short (very distinct in both species). It differs from *Chrysoprasis viridis* Fisher, 1944 mainly by the sides of prothorax not uniformly rounded from anterior to basal margin (rounded in *Chrysoprasis viridis*), and by the metasternum finely punctate (“coarsely foveolate-punctate” in *Chrysoprasis viridis*, according to Fisher 1944). It can be separated from *Chrysoprasis aurata* Aurivillius, 1910 by the sides of the prothorax not uniformly rounded (rounded in *Chrysoprasis aurata*), by the punctures on pronotum and metasternum finer (coarse in *Chrysoprasis aurata*).

All other species of *Chrysoprasis* occurring in Panama belong to the Group *hypocrita*: *Chrysoprasis festiva* Audinet-Serville, 1834; *Chrysoprasis hirtula* White, 1853; *Chrysoprasis hypocrita* Erichson, 1847; *Chrysoprasis rotundicollis* Bates, 1870; and *Chrysoprasis seticornis* Bates, 1880.

As the limits between the Groups *chalybea* and *aurigena* are narrow, and some species can be included in both, we are including *Chrysoprasis birai* in the key for both groups.

In the key to species of the Group *aurigena* the new species can be included in the alternative of couplet “3”, from Napp and Martins (1999) (translated):

**Table:** 

3’(2)	Sides of prothorax not uniformly rounded	***Chrysoprasis birai* sp. n.**
–	Sides of prothorax uniformly rounded and/or longer than wide	**3**

In the key to species of the Group *chalybea* the new species can be included in the alternative of couplet “21”, from Napp and Martins (1997) (translated):

**Table:** 

21’(20)	Sides of prothorax not uniformly rounded from anterior to basal margin; lateral sides of metasternum finely punctate	***Chrysoprasis birai* sp. n.**
–	Sides of prothorax uniformly rounded from anterior to basal margin; lateral sides of metasternum coarsely punctate	**21**

#### Recchia nearnsi sp. n.

Taxon classificationAnimaliaColeopteraCerambycidae

http://zoobank.org/D7C523C4-AD13-4829-AC9D-8DDCF9E20957

**Figures 9–12.**
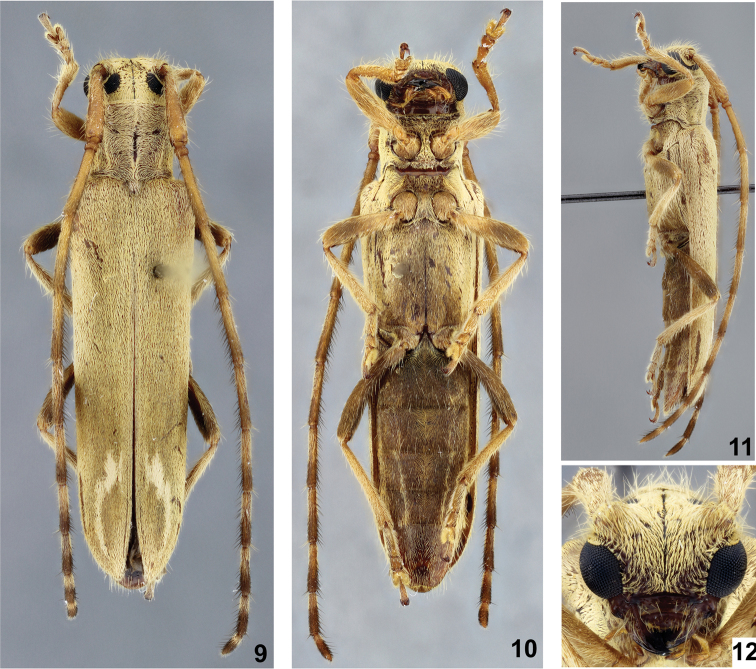
*Recchia nearnsi* sp. n., holotype female: **9** Dorsal habitus **10** Ventral habitus **11** Lateral habitus **12** Head, frontal view.

Figs 9 , 10 , 11 , 12

##### Description.

Female. Integument most orange-brown, with dense, brownish-yellow pubescence.

Head. Frons trapezoidal, abundantly, moderately coarsely punctate; with dense pubescence interspersed with long setae, sparser near clypeus. Clypeus smooth centrally, coarsely punctate laterally. Labrum coarsely punctate. Antennal tubercles finely punctate, with yellowish-orange pubescence. Dorsal surface between antennal tubercles and before upper eye lobes sparsely punctate. Area between middle of upper eye lobes and prothorax, and behind upper eye lobes to about middle of lower eye lobes with dense, yellowish-white pubescence; around margins of eyes with narrow band with yellowish-white pubescence, except for area close to gena. Genae short, sparsely pubescent. Gula glabrous, shiny, coarsely punctate laterally. Coronal suture distinct from clypeus to anterior edge of prothorax. Upper eye lobes with nine rows of ommatidia; distance between upper eye lobes 0.3 times length of scape; distance between lower eye lobes, in frontal view, 0.6 times length of scape. Antennae as long as 1.4 times elytral length, reaching elytral apex at apical end of antennomere IX; orange-brown, with yellowish-orange pubescence; scape densely punctate; antennomeres IV–XI brown on distal portion (this area darker, longer toward distal antennomeres, covering from apical half to apical three-fourths of segment); antennomeres with moderately abundant, short setae on ventral side, interspersed with black, coarse setae (mainly on dark area), denser on antennomeres X and XI; antennal formula (ratio) based on antennomere III: scape = 1.09; pedicel =.0.13; IV =1.0; V = 0.93; VI = 0.86; VII = 0.81; VIII = 0.74; IX =.0.69; X = 0.58; XI = 0,55.

Thorax. Prothorax quadrangular; sparsely, coarsely punctate. Pronotum with pubescence concealing integument; with two narrow, longitudinal, dense, bands with yellowish-white pubescence laterally; with narrow, longitudinal, smooth, shiny area centrally; with band with yellowish-white pubescence centrally, from base to apex, involving smooth area. Prosternum, center of mesosternum, with dense, yellowish pubescence; sides of mesosternum, inner half of metepisterna, center of metasternum with yellowish-brown pubescence (less conspicuous depending on angle of incidence of light). Lateral sides of prosternum, mesosternum and metasternum moderately coarse, sparsely punctate (punctures partially obliterated by pubescence). Elytra elongate, 5.2 times prothorax length, 3.2 times humeral width; integument concealed under pubescence; apical third with three longitudinal bands with dense, yellowish-white pubescence, close each other, together forming a semicircle: innermost short, narrow; central one wider, slightly longer than innermost, with irregular lateral edges; outermost slightly curved, elongate, 1.8 times length of central band, starting near apex of latter, almost reaching elytral apex; elytral apex narrowed, without apical spine. Legs. Segments pubescent, with long, abundant setae, denser, longer on tibiae. Meso- and metafemora more brownish toward apex; metatarsomere I as long as II–III together.

Abdomen. Ventrites I–IV with brownish pubescence, except for wide, yellowish-white band laterally and two narrow, yellowish-white pubescent bands on each side of center (not distinct depending on angle of incidence of light).

##### Dimensions in mm

**(holotype female).** Total length, 15,8; length of prothorax at center, 2.3; widest width of prothorax, 2.3; anterior width of prothorax, 2.1–2.2; posterior width of prothorax, 2.2–2.3; humeral width, 3.8; elytral length, 12.3.

##### Type material.

Holotype female, BOLIVIA, *Santa Cruz*: 4 km N Bermejo (Refugio Los Volcanes, 18°06’S / 63°36’W; 1045–1350 m), 11–17.XII.2012, Wappes & Skillman col. (MNKN).

##### Etymology.

The species is named after Eugenio H. Nearns (Purdue Entomological Research Collection, Purdue University, USA) for his friendship and contributions towards knowledge of Cerambycidae.

##### Remarks.

*Recchia nearnsi* sp. n. is similar to *Recchia ludibriosa* Lane, 1966 but differs as follows: antennae most orange-brown, with short, sparse pubescence; antennomeres IV–XI darker on apical region (more distinct after VII); pronotum with longitudinal, yellowish-white pubescent band; sides of mesosternum, and inner half of metepisterna with yellowish-brown pubescence; elytral length 5.2 times pronotal length; elytra with longitudinal bands with yellowish-white pubescence only at apical third. In *Recchia ludibriosa* the antennae are reddish-brown, with antennomere XI black, and the antennomeres have dense, elongate pubescence, the pronotum is uniformly pubescent, without yellowish-white pubescent bands, the sides of mesosternum and inner half of mesepisterna have dense, long, dark-brown pubescence, the elytral length is 4.0 times the pronotal length, and the apical two-thirds of elytra have oblique whitish bands. It differs from *Recchia ravida* Martins & Galileo, 1985 by elytra with dense yellowish-brown pubescence, obliterating integument, and tree longitudinal yellowish-white pubescent bands at apical third of elytra. In *Recchia ravida* the elytral pubescence is slightly sparser, not obliterating the integument, and has grayish-white pubescent bands on circum-scutellar region and oblique bands on distal two-thirds. Recchia nearnsi differs from *Recchia fallaciosa* Lane, 1966 and *Recchia veruta* Lane, 1966 mainly by the elytral apex not spiny (distinctly spiny in both species), and by the basal half of the elytra without bands of pubescence (present in both species).

*Recchia nearnsi* sp. n. can be included in the alternative of couplet “14”, from Martins and Galileo (1985) (translated; alternative couplet “13” modified):

**Table:** 

13(12)	Upper eye lobes with nine rows of ommatidia; total length 12–16 mm	**14**’
–	Upper eye lobes with five or six rows of ommatidia; total length 7.4–11.3 mm	***Recchia abauna* Martins & Galileo, 1998**
14’(13)	Elytra without longitudinal bands of pubescence on basal half	***Recchia nearnsi* sp. n.**
–	Elytra with longitudinal bands of pubescence on basal half	**14**

## Supplementary Material

XML Treatment for Tropidion birai

XML Treatment for Chrysoprasis birai

XML Treatment for Recchia nearnsi
